# Epidermal Growth Factor Receptor-mutated Lung Cancer as the Initial Manifestation of Germline TP53 Mutation Associated Cancer

**DOI:** 10.7759/cureus.2395

**Published:** 2018-03-30

**Authors:** Surabhi Pathak, Sunny R K Singh, Vatsala Katiyar, Susan Mcdunn

**Affiliations:** 1 Department of Hematology-Oncology, John H Stroger Jr. Hospital of Cook County, Chicago, USA; 2 Department of Internal Medicine, John H Stroger Jr. Hospital of Cook County, Chicago, USA

**Keywords:** li-fraumeni syndrome, lung cancer, tp53, egfr mutation

## Abstract

Epidermal growth factor receptor (EGFR) mutation-driven lung cancer is a rare occurrence in patients with Li-Fraumeni syndrome (LFS) characterized by germline mutations in the tumor protein 53 (TP53) gene. Here we describe a case of primary EGFR mutation-driven lung adenocarcinoma in a young woman with LFS. There is only one other reported case with such presentation. We review the interactions between the TP53 gene and EGFR pathways facilitating lung carcinogenesis. We also review other cases with similar presentations described in the literature and the response to tyrosine kinase inhibitors (TKI) in this rare patient population.

## Introduction

Oncogenic driver mutations in lung cancer vary depending on smoking exposure, gender, and pathologic stage of the tumor. Somatic mutations in tumor protein 53 (TP53) are associated with smoking exposure and advanced disease stage, whereas epidermal growth factor receptor (EGFR) driver mutations are common in women and never smokers [1]. Li-Fraumeni syndrome (LFS) is a rare syndrome of germline TP53 mutation that predisposes patients to a wide spectrum of malignant neoplasms. EGFR mutation-driven lung cancer in patients with LFS is a rare occurrence. Most of the reported cases in LFS patients are secondary EGFR driven lung cancer following treatment for another malignancy (commonly breast) where treatment-related changes are implied in carcinogenesis. Here, we report a patient with LFS who developed primary EGFR mutation-driven lung adenocarcinoma. There is only one other such case reported in the literature [2]. These observations substantiate evidence for cross-talk between TP53 and EGFR pathways which have been noted in preclinical studies of lung carcinogenesis.

## Case presentation

A 28-year-old woman was screened for LFS after her 10-year-old daughter tested positive for germline TP53 gene mutation while undergoing evaluation for newly diagnosed sarcoma. She was found to carry familial heterozygous missense TP53 gene mutation in exon 7 causing nucleotide change c.733G>A with corresponding amino acid change p.G245S in the gene protein. Her two other children from the same partner, aged fourteen and four years, also carried the same mutation. Her partner did not carry the mutation and she had no family history of cancer.

**Figure 1:**
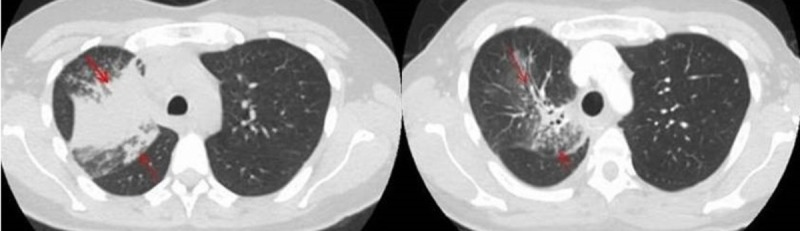
Representative computed tomography (CT) scan images of primary tumor at diagnosis and six months after initiation of erlotinib Left panel is axial CT scan image of the chest with primary tumor in the right upper lobe measuring 4.3 cm x 4.2 cm in size with lymphangitic spread. Right panel is the axial CT scan image of the chest six months after treatment initiation with erlotinib, the right upper lobe tumor now measures 2.4 cm x 2 cm in size consistent with RECIST 1.1 partial response. RECIST 1.1: Response Evaluation Criteria In Solid Tumors, version 1.1

Hence she was enrolled in the high-risk cancer screening program, eighteen months later, she presented with a cough with white sputum and upper back pain. Symptoms did not improve with the course of antibiotics, therefore, she underwent further evaluation. Computed tomography (CT) scan of the chest (Figure 1, left panel) showed a right upper lobe mass. Bronchoscopy and transbronchial biopsy of this mass were diagnostic of lung adenocarcinoma with EGFR exon 19 deletion mutation, clinical stage was T3N2M1, stage IV. Staging positron emission tomography (PET) scan revealed fluorodeoxyglucose uptake in the osteoblastic lesions involving the thoracic spine. Evaluation by neurosurgery deemed the patient not a candidate for surgical intervention, therefore she was treated with external beam radiation therapy of 30 Gray to the thoracic spine in ten fractions for impending cord compression.

As the tumor was EGFR mutated, treatment with erlotinib 150 mg by mouth once daily was recommended. Within two weeks of the treatment, she reported improvement in cough and back pain. Later, she developed flares of moderate cutaneous toxicity (as defined under international EGFR inhibitor dermatological toxicity grading system) to erlotinib. It was treated with the periodic use of topical corticosteroids, topical urea, and two-week courses of doxycycline 100 mg capsules twice daily. Besides cutaneous toxicity, she had no other significant adverse effects from erlotinib. Follow up CT scan six months later showed (Figure 1, right panel) response evaluation criteria in the solid tumor, RECIST 1.1 partial response.

**Figure 2:**
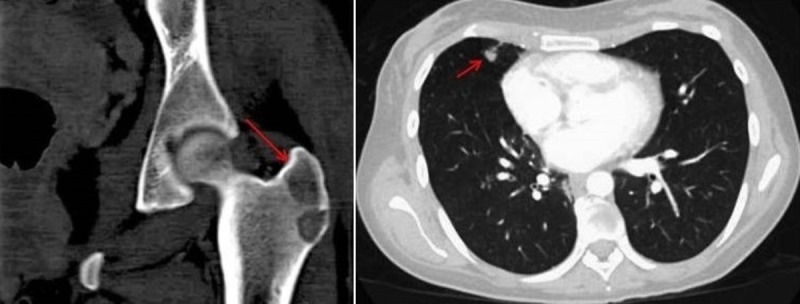
Radiographic evidence of progression at one year from the start of erlotinib treatment Left panel is the representative sagittal section image of computed tomography (CT) without contrast of the left hip showing a new lytic lesion at the base of the left greater trochanter measuring 1.4 cm x 1.3 cm. Right panel is the axial section of CT with intravenous contrast of the chest depicting new lung nodules in the right lower lobe consistent with progressive disease.

Twelve months later she complained of unremitting left hip pain causing her to limp. CT scan of the left hip (Figure 2, left panel) revealed a new lytic lesion. Restaging CT of the chest showed new lung nodules (Figure 2, right panel) consistent with progressive disease. Peripheral blood analysis tested positive for T790M. She was referred for palliative radiation to the left hip while further treatment option with osimertinib in light of T790M positivity was being pursued. 

## Discussion

TP53 gene, also named the guardian of the genome, is the most important tumor suppressor gene encoding p53 transcription factor. Genotoxic stress causes a rise in p53 levels which activates downstream genes for cell cycle arrest, cell repair, and apoptosis. In the absence of normal TP53 protein, DNA damaged cells escape DNA repair or apoptosis, therefore malignant transformation is made possible. The rare LFS is characterized by predisposition to a wide spectrum of malignant conditions. About 70% of families with the syndrome carry autosomal dominant TP53 germline variants [3].

TP53 gene is located on chromosome 17p13.1 and comprises fourteen exons of which ten are in the coding sequence of the 393 amino acids containing protein, one in the non coding exon, and remaining three are alternate exons. It transcribes at least eight different mRNAs and twelve protein isoforms, individual functions of which are still being deciphered. Missense mutations in the DNA binding domain of the protein are the most common alterations in LFS patients followed by nonsense and other mutational subtypes [3]. Differences in predisposition to spectrums of cancers based on mutation sub-type are recently being understood. Missense mutations and mutations with dominant negative effect are associated with early age of tumor onset and high penetrance compared to nonsense and other mutations [4]. Germline and somatic mutations of the gene have similar clinical implications for a given cancer; however, the germline variants are associated with predisposition to multiple primary tumors and treatment-related malignancies [3-5].

EGFR is a transmembrane protein that has multiple ligands including epidermal growth factor and transforming growth factor-α. Ligand binding to EGFR causes an increase in intracellular tyrosine kinase activity and activation of multiple intracellular signaling pathways that promote cellular proliferation [6]. Wild-type p53 binds to the EGFR promoter region thereby increasing its activity. Similarly, tumor-related mutant p53 transcription factor binds to EGFR promoter, however, at a different site than wild-type p53, which leads to disruption in EGFR regulation. Disrupted EGFR regulation can lead to increase in cell proliferation and mutation burden facilitating carcinogenesis [7]. The in-vitro study of lung cancer cell lines suggests that lung cancer cells with TP53 mutations result in over expression of EGFR leading to tumorigenesis [8]. Therefore, we hypothesize that normal lung tissue with germline TP53 mutation in LFS might lead to over expression and mutation of EGFR which then facilitates EGFR mutation-driven lung cancer.

The patient described in our report carried germline TP53 missense mutation with dominant negative effect and developed primary EGFR mutant lung cancer. On literature review, there are two other described cases of primary EGFR mutant cancer in a patient with LFS [2]. There are four other reported cases of secondary EGFR mutated lung adenocarcinoma in LFS patients where treatment-related toxicities were implicated in lung carcinogenesis [2]. Table 1 summarizes known cases of EFGR driven lung cancers in LFS patients, all were in women and never smokers. Most of the mutations are missense (five out of six, one unknown) and all the cases described had at least partial response to EGFR tyrosine kinase inhibitor (TKI) targeted therapy. Duration of response with TKIs in these patients is comparable to historical data with TKIs in EGFR mutant lung adenocarcinoma [9]. Therefore, EGFR targeting appears to be effective in controlling the cancer growth even in the presence of germline TP53 mutation further supporting EGFR as downstream of TP53 pathway.

**Table 1:** Mutational profile of EGFR-mutated lung adenocarcinoma in LFS patients reported in the literature and the response to EGFR TKI therapy All were female. EGFR: epidermal growth factor receptor; TKI: tyrosine kinase inhibitor; RECIST: Response Evaluation Criteria in Solid Tumors; PR: partial response; CR: complete response; PD: progressive disease.

Age at diagnosis (years)	TP53 mutation subtype	Driver EGFR mutation	Previous other cancer	EGFR TKI therapy	RECIST response to therapy
30 (This report)	Missense mutation G245S	EGFR exon 19 deletion	None	Erlotinib	PR (1year)
55	Missense mutation H179Y	EGFR deletion in exon 19	None	Erlotinib	PD
51	Deletion in exon 5	Mutation EGFR L858R	Breast cancer	Erlotinib	PR (1 year)
46	Missense mutation G245S	Mutation EGFR L858R	None (later breast cancer)	Afatinib	CR (1 year)
37	Missense mutation R248W	EGFR exon 20 insertion (A767_S768)	Breast cancer	Lapatinib	PR (6 months)
34	Missense mutation R273H	EGFR deletion in exon 19	Breast cancer	Erlotinib	Not specified
57	Missense mutation R273H	Mutation EGFR L858R	Breast cancer	Erlotinib	PR (1 year)

On the contrary, retrospective studies have reported a negative prognostic value of concurrent somatic TP53 and EGFR mutation and data suggests a poor response to TKI in such patients [10]. Further studies are needed to fully elucidate prognostic significance of concurrent TP53 and EGFR mutations in lung cancer patients.

## Conclusions

In summary, our case highlights the cross talk between TP53 and EGFR in lung tumorigenesis. With increasing number of cases reporting concurrence of EGFR and germline TP53 mutations, it is prudent for treating oncologist to consider LFS when encountering lung cancer in young patients who are never smokers. It appears that EGFR targeted therapy is effective in such patients, however, long-term follow up is needed to determine if concurrent TP53 mutations confer a poor prognosis.

## References

[1] Kawaguchi T, Koh Y, Ando M (2016). Prospective analysis of oncogenic driver mutations and environmental factors: Japan molecular epidemiology for lung cancer study. J Clin Oncol.

[2] Ricordel C, Labalette-Tiercin M, Lespagnol A (2015). EFGR-mutant lung adenocarcinoma and Li-Fraumeni syndrome: report of two cases and review of the literature. Lung Cancer.

[3] Valdez JM, Nichols KE, Kesserwan C (2017). Li-Fraumeni syndrome: a paradigm for the understanding of hereditary cancer predisposition. Br J Haematol.

[4] Bougeard G, Renaux-Petel M, Flaman JM (2015). Revisiting Li-Fraumeni syndrome from TP53 mutation carriers. J Clin Oncol.

[5] Hainaut P, Pfeifer GP (2017). Somatic TP53 mutations in the era of genome sequencing. Cold Spring Harb Perspect Med.

[6] Jorissen RN, Walker F, Pouliot N (2003). Epidermal growth factor receptor: mechanisms of activation and signalling. Exp Cell Res.

[7] Ludes-Meyers JH, Subler MA, Shivakumar CV (1996). Transcriptional activation of the human epidermal growth factor receptor promoter by human p53. Mol Cell Biol.

[8] Vaughan CA, Pearsall I, Singh S (2016). Addiction of lung cancer cells to GOF p53 is promoted by up-regulation of epidermal growth factor receptor through multiple contacts with p53 transactivation domain and promoter. Oncotarget.

[9] Rosell R, Carcereny E, Gervais R (2012). Erlotinib versus standard chemotherapy as first-line treatment for European patients with advanced EGFR mutation-positive non-small-cell lung cancer (EURTAC): a multicentre, open-label, randomised phase 3 trial. Lancet Oncol.

[10] Labbé C, Cabanero M, Korpanty GJ (2017). Prognostic and predictive effects of TP53 co-mutation in patients with EGFR-mutated non-small cell lung cancer (NSCLC). Lung Cancer.

